# On Risk Estimation versus Risk Stratification in Early Prostate Cancer

**DOI:** 10.1371/journal.pmed.1002100

**Published:** 2016-08-02

**Authors:** Sigrid V. Carlsson, Michael W. Kattan

**Affiliations:** 1 Department of Surgery and Department of Epidemiology and Biostatistics, Memorial Sloan Kettering Cancer Center, New York, New York, United States of America; 2 Department of Urology, Sahlgrenska Academy at Gothenburg University, Gothenburg, Sweden; 3 Department of Quantitative Health Sciences, Cleveland Clinic Foundation, Cleveland, Ohio, United States of America

## Abstract

In a Perspective article, Sigrid Carlsson and Michael Kattan discuss Gnanapragasam and colleagues’ accompanying research study on refining risk stratification in early prostate cancer.

Clinically localized prostate cancer (PC) is a heterogeneous disease with highly variable clinical outcome. When counseling a patient with PC, the clinician ought to provide outcome probabilities as accurately as possible, given the patient and data at hand. Here is where the clinical question becomes a statistical one: what is the long-term prognosis and marginal benefit of treatment A versus treatment B versus no treatment—and in relation to death from other causes?

Although randomized trials are under way (e.g., UK ProtecT trial), we do not have data on long-term outcomes comparing radical prostatectomy (RP), radiotherapy (RT), and active surveillance (AS) to provide patients with these numbers. To help clinicians communicate prognostic information and guide appropriate management for the patient, a wide array of risk assessment tools combining clinical and pathologic variables are available [1].

Risk categories typically combine stage, grade, and prostate-specific antigen (PSA) concentration into categorizations such as “low,” “intermediate,” or “high” risk. One of the most commonly used is the D’Amico (1998) risk classification system [2], but limitations include significant heterogeneity, i.e., a wide range in risk of biochemical recurrence (BCR) within each risk group stratum—compared with predicting risk using a mathematical formula—and considerable overlap in risk between the intermediate- and high-risk groups [3]. A modified risk stratification scheme adopted by the National Comprehensive Cancer Network (NCCN) incorporates very low- and very high-risk groups, number of prostate biopsy cores positive, percent cancer core involvement, and PSA density, but is still limited by heterogeneity in recurrence within the risk strata [4]. Recently, novel tissue-based molecular biomarkers have been developed to help sub-stratify risk based on tumor biology [5]. Other means of classifying risk include probability tables, such as the Partin tables [6], which combine variables (stage, grade, PSA) into simple-to-use look-up tables, and risk scores, such as the University of California, San Francisco (UCSF)-Cancer of the Prostate Risk Assessment (CAPRA) score [7], which calculates risk through a summation of points for each variable in a total score of 0–10.

However, risk strata are often collapsed; for example, the Gleason score (GS) is often re-categorized into a three-tiered grouping (6, 7, and 8–10). In addition, because of the range of the scale (from 6 to 10), some patients misinterpret the lowest score (GS 6) as a “middle” score. Communication of risk then becomes an inaccurate reflection of prognosis and could make some patients with low-risk disease opt for primary treatment over initial expectant management. Also, GS 7 is sometimes used as a single score, when 3+4 = 7 or 4+3 = 7 have been shown to be prognostically different; the first number indicates the predominant, or most common, grade, and 4+3 = 7 is consistent with more aggressive disease than 3+4 = 7. Supported by these observations, Epstein and colleagues recently proposed a simplified grading system comprising five grade groups (GG): GS 6 (GG1), GS 3+4 (GG2), GS 4+3 (GG3), GS 8 (GG4), and GS 9–10 (GG5), shown to have strong independent prognostic discrimination for BCR [8].

In this issue of *PLOS Medicine*, Vincent Gnanapragasam and colleagues report on an interesting study using clinicopathologic data for 10,139 men in the United Kingdom to assess risk of prostate cancer-specific mortality. Gnanapragasam and colleagues expanded on the conventional three-tiered “low/intermediate/high” risk strata and developed a novel five-stratum risk stratification system incorporating Epstein’s new GGs [8], clinical stage, and PSA that reflects risk of PC-specific mortality as follows: very low risk (Group 1), low-intermediate risk (Group 2), high-intermediate risk (Group 3), and similar sub-stratification of the high-risk group (Groups 4 and 5) [9]. The authors demonstrated improved predictive accuracy over the three-tiered system [9] both within their study cohort and in an independent validation cohort.

We congratulate Gnanapragasam and colleagues for considering sub-stratification, incorporating the contemporary grading system, and using PC mortality—not BCR—as the endpoint for developing their new risk stratification system, which appears intuitive; if externally validated, systems like this could potentially be clinically appealing for counseling patients. Regarding PSA concentration, however, while most risk tools for localized PC do include this variable, some have suggested that it is not a very strong independent predictor of survival for this patient category [10].

This brings us to a general point about risk stratification versus risk estimation. We are sympathetic to risk grouping systems because they can indeed serve well in clinical practice and guide decision-making, e.g., if very low–low risk, then do not immediately treat; if high risk, then treat. However, heterogeneity within risk groups will still be a limitation, even within a five-stratum system. An alternative or supplementary proposal would be to accurately estimate risk through a mathematical formula, and if groups need to be made for clinical decision-making, the groups could be formed based on the predicted probability scale (e.g., within risk levels).

A generic approach to accurate risk estimation is to develop a multivariable statistical prediction model to calculate the continuous probability of a particular PC outcome and graphically represent the mathematical formula as a nomogram [11]. The conceptual idea is to circumvent the problem with loss of predictive accuracy and power associated with collapsing variables into broad categories, and to extract maximum information in its most granular form and make more efficient use of the available data. Nomograms have been shown to provide superior predictive performance and individualized risk estimations compared to other methods, such as risk-grouping schemata, and to outperform predictions made by opinions of expert clinicians [12].

Several nomograms are available for PC [13] in the form of online computerized risk calculators (e.g., https://apervita.com/community/clevelandclinic and https://www.mskcc.org/nomograms). Nomograms can help clinical decision-making by providing useful information over and above clinical judgment. For instance, while the majority of men with tumors classified as D’Amico “low-risk,” Epstein “GG1,” or Gnanapragasam “Group 1” are likely appropriate candidates for conservative management, and, conversely, the majority of men with D’Amico “high-risk,” Epstein “GG5,” or Gnanapragasam “Group 4/5” PC are likely to be recommended treatment, the decision to treat or not to treat is always a clinical judgment call—one that needs to take into account a man’s general health and life expectancy in discussions with the individual patient. Is the patient young or old? Is he fit for curative treatment or does he have comorbidities? The NCCN guidelines for PC [14], among others, make differential treatment recommendations based on expected patient survival (life expectancy).

The statistical question thus becomes: what is the long-term risk of PC mortality with treatment compared to risk of death from other causes? A pre-RP nomogram predicting long-term risk of PC death (https://www.mskcc.org/nomograms/prostate) can provide useful information in the following way: “This number shows, as a percentage, your probability of surviving PC for 10 years following RP. This probability means that for every 100 patients like you, X will survive PC and Y will have died from PC.” Based on the observation that few, if any, valid or clinically useful tools for measuring life expectancy exist, Kent and co-workers recently developed and validated a prediction model for other causes of mortality in patients with localized PC [15], which takes into account age and comorbidities and can provide an estimate of the 10–15-year risk of death from PC if untreated and in relation to death from other causes. Of course, the validity of such methods will depend on the robustness of their validation and the relevance of the underlying data to each individual patient in terms of the parameters included and population applied to. As such, estimates from such methods to aid treatment decision-making need to always be used in conjunction with sound clinical judgment.

Technological advancements allow for nomograms to be integrated into the electronic medical record and used directly in patient–clinician consultations, and can incorporate continuously updated collected data from a large number of patients into dynamic predictive modeling. In this way, provision of accurate risk estimations through the use of nomograms can be useful in clinical decision-making as supplements to risk grouping systems.

## Abbreviations

AS: active surveillance
BCR: biochemical recurrence
CAPRA: Cancer of the Prostate Risk Assessment
GG: grade groups
GS: Gleason score
NCCN: National Comprehensive Cancer Network
PC: prostate cancer
PSA: prostate-specific antigen
RP: radical prostatectomy
RT: radiotherapy
UCSF: University of California, San Francisco
